# Negotiating Mental Health and Professional Identities: An Interpretative Phenomenological Analysis of Nursing Students’ Categorisation, Disclosure, and Support-Seeking

**DOI:** 10.3390/bs16101922

**Published:** 2026-10-16

**Authors:** Eddieson Pasay-an, Analita Gonzales, Jeneth Gutierrez, Abdulellah M. Alsolais, Reem Humaidi Alalawi, Amal Ali Alharbi, Nuha Ayad H. Alatawi, Mathar Mohideen Nagoor Thangam, Sumathi Robert Shanmugam, Abdulrahman Sulaim Almutairi, Sahar Hamdy El-Sayed, Wireen Leila Dator

**Affiliations:** 1 Nursing Administration and Education Department, College of Nursing, King Khalid University, Abha 61421, Saudi Arabia; 2 College of Nursing and Allied Health Sciences, St. Paul University Manila, Manila 1004, Philippines; 3 Nursing Administration and Education Department, Faculty of Nursing, University of Tabuk, Tabuk City 71491, Saudi Arabia; 4 College of Applied Medical Sciences, Shaqra University, Al Quwaiyah 11971, Saudi Arabia; 5 Medical and Surgical Nursing Department, Faculty of Nursing, University of Tabuk, Tabuk City 71491, Saudi Arabia; 6 Department of Maternity and Pediatric Nursing, College of Nursing, Princess Nourah bint Abdulrahman University, Riyadh 11671, Saudi Arabia

**Keywords:** mental health identity, nursing students, interpretative phenomenological analysis, social identity theory, symbolic interactionism, identity negotiation theory, stigma, seeking support, Saudi Arabia

## Abstract

**Introduction:** This study explores how students develop, negotiate and represent their mental health alongside their professional identities. The aim is to understand how nursing students construct and negotiate their mental health identities in academic and clinical environments. **Method:** Using Interpretative Phenomenological Analysis (IPA), this qualitative research employed nine nursing students enrolled in the Bachelor of Science in Nursing (BSN) programme at King Khalid University in Saudi Arabia. Semi-structured interviews were conducted in Arabic, the participants’ native language, and professionally translated to English for analysis. Interviews were analyzed using a six-stage IPA protocol and an integrated coding framework operationalizing concept from Social Identity Theory, Symbolic Interactionism, and Identity Negotiation Theory. **Results:** The data led to five themes: (1) Navigating the Stigma Boundary: The ‘tough nurse’ imperative, strategic silence or selective disclosure as identity management. (2) Developing the ‘Mental Health’ Label: Repertoires and levels of surveillance/monitoring. (3) The Clinical–Classroom Divide: Contexts affecting identity performance and role transitions. (4) Help-Seeking as Identity Betrayal: The cost to identity of seeking formal help and preference for informal support. (5) Reconciling Personal and Professional Identities: Future self-prototype construction, divergent personal–professional developments, and post-trauma story-telling. **Conclusion:** Nursing students’ mental health identities are socially constructed and strategically negotiated across contexts. This multi-theoretical model advances understanding beyond deficit-oriented frameworks, showing that help-seeking is limited by identity barriers.

## 1. Introduction

A growing concern exists for the mental health of nursing students. Across empirical studies, nursing students have been shown to be at higher risk than the average university student for experiencing symptoms of psychological distress. These include anxiety, depression, and burnout (Mitchell, 2018; Galbraith et al., 2014). Nursing students are exposed to a uniquely stressful curriculum environment consisting of heavy course loads, rigorous clinical placement experiences, and the emotional demands of providing patient care (Happell et al., 2023). Research has also indicated that while many nursing students struggle with these challenges, they tend to underutilize support services. The majority of students cite fear of disclosing their mental health struggles as the most common reason for not seeking help. They are concerned that disclosure will affect their ability to become a nurse (Mitchell, 2018; Galbraith et al., 2014).

Students in nursing programs develop and articulate their professional identities through ongoing participation in both educational classrooms and clinical settings. In these environments, students continually engage in negotiations regarding competing discourses. These relate to competency, stoicism, and self-care (Patterson et al., 2017; Patterson et al., 2018). During this process of professional socialization, nursing students define, represent, and interpret their own mental health status. A process significantly influenced by institutional expectations and shaped by perceptions of what peers, faculty, and clinical supervisors expect of them (Sukhera et al., 2022). These constraints on self-disclosure may limit students seeking help and accessing institutional support services (Galbraith et al., 2014; Mitchell, 2018). Studies examining stigma within medical education indicate that two competing discourses exist. These are normalizing disclosure versus pressure to perform, and both influence whether students are willing to seek help (Sukhera et al., 2022). Furthermore, studies have demonstrated that even when discourses of heroism and resilience are used to normalize help-seeking, institutional power dynamics continue to serve as barriers to disclosure (Sukhera et al., 2022).

The Saudi Arabian environment is culturally unique. It forms an important backdrop to the ways in which these forces interact. Mental health stigma exists within conservative social norms of Saudi society (Alluhaibi & Awadalla, 2022). When people experience psychological distress, it is usually understood through religious or family-based frameworks, with medical models of illness less commonly used (Al-Subaie et al., 2020; Altwaijri et al., 2019). Historically, nursing education in Saudi Arabia began as technically oriented, hospital-based diploma programs administered by the Ministry of Health and other government bodies, with university-level programs introduced only in the mid-1970s; psychosocial support has not traditionally been a curricular emphasis (Aljohani, 2020). The most recent healthcare reform, Vision 2030, has created additional pressures on nursing students, who must quickly adapt to changing professional expectations. Further, because the nursing profession is primarily staffed by females, cultural constraints to maintain modesty and protect family honor can create barriers to seeking help from university professionals and deter students from disclosing mental health concerns (Alluhaibi & Awadalla, 2022). This combination of environmental factors makes Saudi Arabia a significant setting for understanding how nursing students’ professional identities develop and how those identities relate to their mental health identities. The Social Identity Theory (SIT), developed by Tajfel and Turner (1979), is a theoretical model that allows researchers to study how nursing students categorize themselves into groups based on their similarity or dissimilarity to other members regarding their experiences with mental health. As such, SIT allows us to understand how mental health becomes a prominent category of identification that influences the behaviors and attitudes nursing students exhibit toward one another and whether they disclose their own mental health difficulties to others (Haslam et al., 2009).

Symbolic Interactionism, which includes theories from Mead (1934) and Blumer (1969), is another theoretical model that researchers use to explore how nursing students create interpretations of what it means to experience distress. By studying how nursing students perceive their distress and develop meanings related to it, we can better understand how they present themselves to others—as experiencing or not experiencing distress (Sukhera et al., 2022).

The Identity Negotiation Theory, also known as Intercultural Communication Competence, was developed by Ting-Toomey (1986, 2005), who defines identity negotiation as the process of negotiating one’s identities in different environments or roles. For example, a nursing student who experiences anxiety or depression must simultaneously navigate the role of nursing student and manage her mental health difficulties. As such, researchers using the Identity Negotiation Theory can investigate how changes in context affect students’ decisions about whether to disclose their mental health issues and whether to seek help (Ting-Toomey, 2005). The Integrating Social Identity Theory and Symbolic Interactionism provides insight into the processes of constructing mental health identity, while combining these two theories with the Identity Negotiation Theory illustrates the performance of those constructed identities across different settings.

To date, no comprehensive study has used all three theories—SIT, Symbolic Interactionism, and the Identity Negotiation Theory—in conjunction to examine how nursing students develop a sense of mental health identity. Therefore, we believe that our study provides a unique opportunity to further understanding of how nursing students think about and experience their mental health in tandem with their developing professional identity.

This study examines the interconnections among stigma, help-seeking, mental health identity, and professional socialization—areas that have been addressed separately but not simultaneously in prior research. Thus, our research is the first to explore how students’ perceptions of their peers influence their decisions to seek help for mental health issues, representing a significant step forward in understanding how social interactions affect mental health behaviors. It fills a gap in the literature by using a multiple-lens theoretical model that examines how nursing students develop a sense of their mental health status alongside their developing professional identity. It also examines how nursing students negotiate their professional identity and how contextual transitions affect help-seeking behaviors.

Using Interpretative Phenomenological Analysis (IPA) this study goes further than simply describing what happens during the process of becoming a nurse. It explains how nursing students make meaning based on their professional and personal identity (Eatough & Smith, 2017; Smith et al., 2009). An integrated coding framework operationalizes concepts from each of the three theoretical models studied. This demonstrates transparency in terms of theoretical fit, cross-lens convergence, and tension. These elements contribute to a more comprehensive and methodologically robust explanation of how mental health status evolves as a social–professional concept within nursing education. Advancing these theoretical models, in turn, allows recommendations to be developed, including stigma-reduction interventions, mental health literacy programs, and policy changes that promote safe help-seeking behavior and authentic professional growth.

## 2. Methods

### 2.1. Study Design

This research used Interpretative Phenomenological Analysis (IPA). IPA is a qualitative methodology focused on understanding how individuals make sense of their significant life experiences (Smith et al., 2009). We employed the double hermeneutic. We made sense of how our participants constructed meaning about their own mental health. We also examined its connection to the stressors related to becoming a nurse. With IPA, we could go beyond simply describing the nursing students’ experiences. We gained insight into how they create and interpret meaning for themselves. This is based on their professional and personal identity (Eatough & Smith, 2017). This study adheres to the Consolidated Criteria for Reporting Qualitative Research (COREQ) guidelines (Tong et al., 2007).

### 2.2. Participants and Setting

Data collection occurred at the King Khalid University (KKU) College of Nursing in Saudi Arabia. Nine nursing students were recruited using purposeful, homogeneous sampling. All participants were enrolled in the BSN program at levels 4–8.

The selection criteria included: (1) current enrollment in the BSN program at KKU; (2) experience of a mental health issue (anxiety, burnout, or emotional distress) that impacted academic and/or clinical performance; and (3) willingness to participate in a lengthy, reflective dialogue concerning their experiences.

Recruitment took place from May to June 2026. Potential participants were identified through an announcement in the cohort WhatsApp group, and initial screening was completed by the researchers in person. Students who met the eligibility criteria were provided with the information sheet and consent form. Of the 15 students approached, four declined to participate and two did not meet the eligibility criteria; the remaining nine students consented and completed interviews. Recruitment and eligibility screening were conducted independently of course instructors and clinical evaluators.

An upper limit of 11 participants was set prior to recruitment. This allows adequate data for complete analysis while maintaining the thematic detail and focus necessary for IPA (Smith et al., 2009). The researchers used Guest et al.’s (2006) determination of thematic saturation as a guide. Saturation is defined as obtaining enough information that no further themes or codes are generated from successive interviews. No new themes or codes emerged from the ninth interview, and its content confirmed existing patterns rather than introducing new ideas; saturation was therefore judged to have been reached, and recruitment ceased at nine participants.

All participants were female. This reflects the predominantly female nursing student population in Saudi Arabia. Levels correspond to academic years in the BSN program (Level 4 = third year; Level 8 = final year). Self-described experiences reflect participants’ own language during screening. They were not clinically verified.

### 2.3. Data Collection Procedure

Data collection occurred using one-on-one, in-depth, semi-structured interviews. Interviews were organized around an initial conceptual framework which directed the interview process via open-ended questions allowing participants to respond to questions verbally. Participants provided narrative responses relating to their experiences, therefore providing insight into how they constructed their identities. Examples of some interview questions include: “What does ‘mental health’ mean to you as a nursing student?”; “How did being a nursing student affect your views of mental health?”; and “Provide an example of when you were doing clinical practice and you believed your mental health either limited or uniquely contributed to your perceptions.” Interviews were structured as phenomenological conversations where researchers worked collaboratively with participants to create meaning together. Researchers utilized probes such as: “You mentioned earlier, can you explain/expand on that?” and “Why does this experience relate to your vision of self as a future nurse?” Researchers conducted all interviews privately, obtained written consent to record, and arranged for professional transcription of all audio recordings. Interviews were conducted by two female researchers with qualitative interviewing experience and training. None of the interviewers was responsible for assessing, grading, or supervising the participants in the current academic term. Before the interview, participants were reminded that refusal or withdrawal would not affect grades, clinical evaluation, or standing. Audio files were transcribed by a professional service under confidentiality agreement. Transcripts were de-identified by removing names, locations, dates, and other identifiers; pseudonyms were assigned and files were stored on password-protected/encrypted devices accessible only to the research team.

Interview length ranged from 45 to 90 min (Mean = 62 min). All interviews were completed in Arabic, the participants’ native language. Transcription of the audio recording took place in Arabic. A professional transcription company produced an initial transcription of each audio file in Arabic. Translations of each Arabic transcript to English were completed by a bilingual researcher proficient in speaking both languages. Each translation underwent a back-translation by an independent bilingual researcher proficient in speaking both languages. The original Arabic transcript and the back-translated Arabic transcript were reviewed for semantic equivalence by a bilingual review team member. Discrepancies were resolved through team discussions. This process-maintained research integrity throughout translation, ensuring no loss of interpretive meaning during cross-cultural transfer. The interview guide was reviewed by the research team and refined for clarity and flow after the first interview; no formal pilot test was conducted.

### 2.4. Data Analysis

As outlined by Smith et al. (2009), our analysis proceeded according to the six-stage IPA analytical process, which are as follows:

Stage 1—Read and Re-read: The researchers fully immersed themselves in the raw data through multiple readings of the transcripts.

Stage 2—Initial Noting: In an effort to identify potential avenues of inquiry, we used free association techniques to engage with the semantic content of the data and wrote comments and marginalia in the transcripts.

Stage 3—Develop Emerging Themes: We read and reread the transcripts, noting areas that seemed worthy of additional investigation. We then abstracted these observations to begin developing preliminary themes.

Stage 4—Identify connections between emerging themes: Once we had developed some preliminary themes, we sought to determine if there were connections between them. To this end, we abstracted and synthesized the themes to form cohesive thematic clusters.

Stage 5—Move on to the next case: To allow us to approach each new participant with an open mind, we “bracketed” our existing understanding of the data from previous participants. This allowed us to view each participant’s experiences in isolation rather than viewing each participant through the lens of what we knew from previous participants.

Stage 6—Look for patterns across cases: Through iterative review of all the cases, we determined if there were commonalities across participants’ experiences represented at a higher level of abstraction. When a commonality was identified across participants’ experiences, we further investigated it through metaphors, temporal reference points, and importing other theoretical lenses to inform interpretation.

This multi-step process facilitated movement from individual-level case analysis toward identification of common patterns across participants’ experiences while maintaining the idiographic richness associated with IPA (Smith et al., 2009; Eatough & Smith, 2017).

### 2.5. Ethical Consideration

The study was conducted using an Institutional Review Board-approved protocol by the King Khalid University Research Ethics Committee. Approval number: KKU/REC/2024/089; date of approval: 15 August 2024.

An Informed Consent Form was used to provide all participants with their rights as volunteers, including the right to withdraw without penalty. A Protocol for Supporting Participants’ Mental Health Needs was developed due to the sensitive subject matter being explored. Referral paths to university-based counseling services were included in this protocol.

### 2.6. Reflexivity

All members of the research team are faculty at different universities. Thus, there is potential for power dynamics and social desirability bias created through these professional roles. The researchers employed ongoing reflexive bracketing. They held bi-weekly debriefing sessions throughout the project. These sessions discussed how assumptions about teaching and learning may have influenced their understanding of the data. They also addressed biases related to pedagogy. Additionally, they examined the influence of institutional context on their interpretation of the data. These meeting notes were recorded in a group research journal. This allowed assessment of each researcher’s contribution to early interpretations.

Social desirability bias was further mitigated by assuring participants that their participation would not affect their evaluation as students. Interviews were structured as phenomenological discussions. This created distance from the educators’ identity. It also established an empathic relationship through active listening. Familiarity with nursing curricula was viewed as a way to build rapport, rather than limit it.

Member checking was done with three participants (Aisha, Maryam, and Zahra). This ensured that the researchers’ interpretations accurately reflected what they experienced during the interviews. Members checked whether the researchers accurately represented their experiences and perceptions of their undergraduate nursing program.

### 2.7. Credibility

Credibility of this study was determined by the researcher’s engagement in prolonged data collection; the use of peer debriefing with members of the research team; and member checking with three participants to review preliminary summaries of thematic categories.

Transferability of findings was increased due to the extensive descriptions provided of the participants and the research site(s) where the data collection took place.

Dependability of results was achieved by maintaining an audit trail of all analytical decision-making processes, coding changes, and team discussions.

Confirmability of findings was also maintained through reflexive journaling and triangulating interpretation among multiple researchers (Lincoln & Guba, 1985).

## 3. Results

Table 1 presents the anonymized characteristics of the nine participants: three were in Level 4, two in Level 6, and four in Level 8, with prior clinical experience ranging from 6 to 18 rotations.

The process of analyzing the data used the six-stage IPA protocol (Smith et al., 2009). This resulted in five superordinate themes representing the ways in which nursing students developed, negotiated, and expressed their mental health identity in both academic and clinical environments while completing their nursing education. An integrated coding framework using theoretical codes linked each theme to concepts derived from the Social Identity Theory (SIT), Symbolic Interactionism, and the Identity Negotiation Theory. The use of this framework allowed for explicit documentation of both cross-lens convergence and divergence. The combined display of superordinate themes and their corresponding theoretical maps can be found in Table 2.

### 3.1. Theme 1: Navigating the Stigma Boundary

Participants’ views of mental health stigma in nursing education and ways of managing their identity borders to prevent negative social categorization when they experienced emotional disturbance (Goffman, 1963). There was considerable agreement among all three lenses regarding the nature of each of the subthemes described below. However, each lens offered additional information on this theme than the other two lenses combined.

*Subtheme 1.1: The “Strong Nurse” Imperative*

The participants noted that mental health stigma existed in their nursing cohort, where students who experience emotional disturbances may not have the skills necessary for them to perform their duties as competent nurses. Participants also stated that this cohort model was used to create a boundary between a “strong” nurse and one who may be perceived as vulnerable or less able to deal with stressful situations.

“…this [mental health] stuff is the opposite of what we are as future nurses. We hold people together. If you can’t do that then you’re going to be viewed negatively like maybe you aren’t cut out to be a nurse.”

(Fatima, Level 4)

“…a ‘strong’ nurse means you take care of the suffering, of yourself and the patient without allowing the suffering to affect your outside presentation… you are not simply ‘stressed’, you fail a spiritual test.”

(Maryam, Level 6)

“The preceptor had once mentioned that nurses are the backbone of hospitals. She explained to me that patients could sense fear and uncertainty. From that time on, I always hide how I felt, even though I did so internally. This is what it meant to be a nurse according to me.”

(Maha, Level 8)

These participant quotes illustrate both group norm reinforcement and the possible danger involved in being classified outside of the group. The quotes illustrate a particular group prototype, with emotional stability as an important aspect of a nursing identity, and potential results of not aligning with this prototype. The Social Identity Theory illustrates how these meanings exist and continue through peer observation and implied judgment. The quotes illustrate the looking-glass self-process which is at the heart of Mead’s symbolic interactionism theory. Moreover, the Identity Negotiation Theory supports an alternative way of maintaining identity cohesiveness across contexts by fulfilling the expectations of the various roles (i.e., student, future nurse, colleague), by using the dominant prototype.

*Subtheme 1.2: Silence as Self-Protection*

The following excerpt contains participant quotes illustrating the ways students used silent behavior as a form of social control to maintain their identities as “the competent student nurse,” particularly within the confines of observable academic and clinical environments.

“Regardless of whether I trust them or not…I would rather keep it on myself or inside of me so it can remain protected because when I say it loud, it become the reality I mean”

(Huda, Level 6)

“I have learned to become quite skilled at concealing myself. When we are in the skills lab, and everyone is practicing injections and I am experiencing a panic attack internally, no one recognizes that. I smile, I nod, I complete the procedure. However, inside me, I am completely crumbling. If I will tell to my group, it will make my confidence low which is not good for my future.”

(Maryam, Level 6)

This quote provides evidence of participants’ use of self-protection through avoiding the use of identity marker, mental health, and thereby preventing herself from being labeled into the out-group. The symbolization of silence demonstrates the way in which participants manage their presentation of self in order to protect their identity as “the competent student nurse” in relationships with peers and instructors. The statement by Huda, “once you say it out loud, it will become reality”, illustrates the performative aspects of identity; the act of giving a name to an experience result in its transformation from private experience to public identity classification. Maryam expands upon this by depicting the deliberate efforts involved in concealing oneself during the performance of clinical skills procedures, and illustrates that silence is not simply inactive, but requires considerable cognitive and emotional energy to achieve. By utilizing silence as a means of limiting exposure costs and therefore preserving identity integrity, participants illustrate a cross-contextual legitimacy strategy as outlined by the Identity Negotiation Theory.

*Subtheme 1.3: Selective Disclosure*

Most students practiced extensive non-disclosure of their identities. All articulated selective disclosure of their identities to select peers, indicating a high degree of sophistication in identity boundary management practices.

“There is just one person that I have told about my situation. She understands. We’re at the same level. But I asked her to promise not to tell anybody else. It’s like I want to have somebody that knows about me, but I’m going to deny that aspect of me to everybody else. So, I pick and choose the people that get to see that part of me.”

(Noura, Level 8)

“I told my friend because she too in the same school and she has confided in me some of her own struggles. It was mutually understood. But I would never share anything like that with my clinical group because they are competitive and I do not know who would use that information against me during evaluations.”

(Huda, Level 6)

In these examples, the Social Identity Theory illustrates strategic in-group boundary management: the students created mini-in-groups (the trusted friend or roommate), providing opportunities for limited identity expression while maintaining the “strong nurse” image to the out-group. Moreover, Symbolic Interactionism illustrates the dyadic meaning-making process—the friend or roommate “gets it”, due to their shared interpretive frames developed from similar experiences as nursing students. Noura illustrates the calculated risk assessment associated with selective disclosure: evaluating the benefits (mutual support) versus risks (competitive utilization in clinical evaluation) associated with revealing personal characteristics. Lastly, the Identity Negotiation Theory illustrates the contextually dependent determination of selective disclosure: determining what legitimate gains (e.g., emotional support) versus legitimate losses (loss of identity value) are realized by selectively revealing personal characteristics and selecting a compartmentalized approach.

In the context of point of convergence, all three lenses converge on the pervasive effect of stigma on students’ formation of mental health identities. The Social Identity Theory illustrates group-based standards influencing the construction of mental health identities; Symbolic Interactionism illustrates how meanings are interactively constructed; and the Identity Negotiation Theory illustrates how students strategically manage boundaries between disclosing and withholding disclosure in multiple contextual relationships. A possible area of disagreement is whether there is a primary agent; the Social Identity Theory and Symbolic Interactionism emphasize the role of structural/interactions, while the Identity Negotiation Theory emphasizes individual deliberation; a divergence representative of larger debates surrounding the identity theory.

### 3.2. Theme 2: Developing the “Mental Health” Label

This theme looks at how students assign meaning to their emotional pain and represents variation in how students construct identities using narrative and linguistic categorizations to define their experiences.

***Subtheme 2.1: Medical* vs. *Moral Framing***

Participants used varied frames when describing psychological distress. Student accounts varied in terms of frames used to describe their psychological distress, and there were also different effects on identity development based on these frames. For instance, participants mentioned the following:

“I would never think to say that I suffered from a ‘mental illness’. I am simply under a lot of stress. We all are. If I had described my experience using the terminology related to depression or some other type of mental illness, then I believe I would have felt…I don’t know…too much drama? Too serious about this long-term thing? I am simply going through an unfortunate period in my life right now.”

(Aisha, Level 4)

“I finally went to see my doctor and he informed me that I had anxiety. Once I knew that term, it really helped. Before I learned this term, I believed that I was simply weak. Learning that anxiety is a medically identified condition allowed me to see things differently concerning myself than I had previously.”

(Zahra, Level 8)

“The first time that I experienced these feelings, I believed it was due to lack of prayer or because I was being punished for something. I never considered it could be a medical condition. My mom has always said that having a strong faith will keep you safe from sadness. Therefore, I believed my faith was weak.”

(Bayan, Level 4)

These quotes illustrate Symbolic Interactionism in action. In the first quote, Aisha utilized a normalizing frame to prevent the medical classification of stress so that she retains control of her identity (“stressed” is temporary; “depressed” has potentially negative implications for identity). In the second quote, Zahra employed a medicalization frame that changed her moral weakness into a biological condition allowing herself compassion and rebuilding her identity. Moreover, Bayan’s account exhibits the moral/religious framing many students use at first to attribute psychological pain to spiritual shortcomings rather than medical conditions.

The Social Identity Theory describes the possible types of group prototypes through comparisons: the first student avoids being labeled “mentally ill” by the out-group; the second student labels a category itself rather than avoiding it. The Identity Negotiation Theory indicates that students struggle with identifying themselves with varying labels depending upon context: for example, while Aisha may use “stress” to produce solidarity among other students during casual social interactions, she might have trouble utilizing this label when conversing with medical professionals who require greater levels of performance.

***Subtheme 2.2: Self-Surveillance and Monitoring***

Students expressed that they constantly monitored themselves to recognize and regulate their psychological states before they could reveal them to others. Participants mentioned the following:

“I monitor myself all the time. Before going to the clinic I ask: Am I too anxious? How much anxiety can I hide? Is there anything my Preceptor can see? It seems like I have two me’s: one that experiences emotions, and another that observes to make sure no one else notices the first.”

(Bayan, Level 4)

“In every clinical rotation, I go in front of the mirror and practice my face. I look at my hand for tremors. I think about how I will answer if someone says ‘Are you alright?’ It’s become something I do now. I’m my own inspector before anybody else inspects me.”

(Reem, Level 8)

The quotes above illustrates reflexive self-monitoring (Symbolic Interactionism) in that students have incorporated the generalized other of the nursing profession into themselves, therefore surveying themselves through the lens of this other. Reem describes the pre-clinical ritualization of self-surveillance, transforming internal surveillance into observable behavior preparation. The Social Identity Theory illustrates how identity boundary maintenance functions of self-surveys as “the watcher” self defends the “nurse-student” self from being categorized as non-group. Moreover, the Identity Negotiation Theory illustrates the split self-process of developing identities between contexts: students develop the need to have consistency between the “self-experiencing” and “self-performing” with regard to negotiating the differences in role expectations across contexts.

The Social Identity Theory and Symbolic Interactionism converge on social constructs of mental health categories—whether “stress”, “anxiety”, or “burnout”, categorizations are not neutral and hold identity consequences. Identity Negotiation Theory creates conflict by illustrating that label utilization depends on context: students utilized different labels in different contexts demonstrating that mental health identity is unstable and based on the interaction taking place.

### 3.3. Theme 3: The Clinical–Classroom Divide

The clinical–classroom divide is an area of significant variation among students when they experienced and managed their mental health identities; it provides examples of two different identity domains—one which includes clinical settings and the other includes classroom settings.

***Subtheme 3.1: Performing Competence at the Bedside***

Students believed that they had to demonstrate competence in emotionally regulating themselves during clinical experiences. Clinical experiences provided an environment for students to experience a high-risk identity domain due to the interpersonal characteristics of bedside interactions.

“If you are crying while taking care of patients, you fail not only yourself, but you will also fail your patients. For that reason, I show that I am strong, I take on the role of the nurse that the patients need, whether or not I am crumbling within.”

(Maryam, Level 6)

“…during my pediatric rotation, I had a patient whose likeness to my late younger brother triggered me. I wanted to cry so terribly, but I understood that if I did, the nurse instructor would deduct points for lack of professionalism. Thus, I went into the supply closet, took three deep breaths and then returned with a smiling face. The patient never knew.”

(Fatima, Level 4)

The Identity Negotiation Theory is primarily responsible for explaining this phenomenon. Participants report changing roles into that of a ‘clinical nurse’ and describe the legitimate expectations required of this role. The ‘mask’ metaphor exemplifies Goffman’s (1959) concept of front-stage performance required for clinical settings. Moreover, Fatima’s account shows the specific situational factors that necessitate emotional suppression and describes the tactics used to preserve professional composure. The Social Identity Theory highlights how professional identity varies depending on context: the in-group prototype of “competent nurse” is most salient in clinical areas, whereas the identity of “struggling student” is suppressed. Symbolic Interactionism demonstrates that the interactional components create risk: the provision of patient care presents the greatest risk for assessing professional identity and failure to comply may result in injury to both self-identity and patient safety.

***Subtheme 3.2: Vulnerability in the Classroom***

Unlike clinical settings, the classroom offered slightly greater latitude for students to show emotion and expressed some types of emotions as acceptable given certain parameters.

“At school, I sometimes tell my friends about having a bad day. Even though, I do have to be careful about what I say so I won’t be caught by an instructor or so that another student thinks that I can’t handle stress… It’s still somewhat dangerous. But it’s safer than in hospitals. At least in school, we’re all students together.”

(Maryam, Level 6)

“During our mental health lecture, the teacher asked if anybody had experienced anxiety. I desperately wanted to raise my hand because I have been experiencing anxiety for two years now. But I looked around and no one else was raising their hands, therefore neither did I…the classroom seemed safer than the hospital, however not safe enough.”

(Aisha, Level 4)

In this case, the Identity Negotiation Theory illustrates how contextual variance affects norms of disclosure: classrooms offer more opportunities for vulnerability since role expectations are less stringent but students recognize possible identity threats from teachers. Aisha’s account illustrates the paradox of the classroom as theoretically safer space yet pragmatically constrained by peer observation and fear of being the first to disclose. The Social Identity Theory provides insights into why in-class peer in-group solidarity (“we’re all students together”) allows for some level of disclosure but requires students to remain cognizant of visibility and develop mechanisms to regulate visibility. Symbolic Interactionism illustrates the necessity of watchful interactional behavior in controlling disclosure.

***Subtheme 3.3: Identity Fragmentation During Transition***

Participants found transitioning between the clinical environment and the classroom to be particularly confusing. Consequently, they needed to rapidly change their identities. Participants felt these transitions placed an excessive burden on their mental health resources.

“I think that is the most difficult part. The transition…sometimes I am in school and I feel somewhat normal. I talk to my classmates. Other times, I am at the hospital and I need to become an entirely new person. Some days I can barely tell if I am really being who I am.”

(Huda, Level 6)

“For me, the most difficult part of my day is getting on the bus after class and going home. Those twenty minutes are where I have to shift from ‘student mode’ to ‘nurse mode.’ I get dressed in my scrubs, adjust my makeup, and leave all other aspects of myself behind. When I arrive at the hospital, I am no longer who I used to be. And when I go back to school, I do not know how to shift back again. I feel lost.”

(Fatima, Level 4)

These two quotes illustrate the identity continuity problem or discontinuity that is a core aspect of the Identity Negotiation Theory. The students had role conflicts between “student self,” “clinical self” roles, and the transition caused identity fragmentation. Fatima added the dimension of time and place to describe the transition experience: she described her commute as a transitional zone (liminal space) in which she transformed her identity.

The Social Identity Theory explained little about this sub-theme since the in-group prototype remained constant and the participant demonstrated variable enactment of the prototypes based on context. Symbolic Interactionism did capture the biographical disruption; however, the participants’ personal narratives lacked the ability to include their rapid changing of identities within their life stories. This created an existential dilemma (“what is the authentic version of me?”).

### 3.4. Theme 4: Help-Seeking as Identity Betrayal

This theme describes participants’ ambivalence towards obtaining formal and informal types of support for managing stress related to their participation in the research. Although participants’ views regarding seeking help do reflect a desire to gain access to resources and resolve problems, they primarily represent participants viewing seeking help as potentially harmful to their professional identity.

***Subtheme 4.1: Perceived Threats to Future Professional Suitability***

The strongest deterrent to participants pursuing assistance was the perception of threats to their future professional suitability.

“… at times, I am asking myself what if employers saw it and think I am unable to handle stress? I’d rather suffer now than risk my future.”

(Huda, Level 6)

“My cousin is a nurse working for the government, and she told me that in the hiring process, they will review all records. She stated that any record of mental health treatment is a huge red flag.”

(Aisha, Level 4)

Through applying the Identity Negotiation Theory, we see how participants use fear of breaking group norms as defined by the “Strong Nurse Prototype” to avoid seeking assistance.

By doing so, participants are excluded from their peer group as well as their profession. Additionally, Aisha shows the intergenerational transmission of stigma in the profession. As a result of receiving career advice from a successful nurse, she reinforced the idea that there are risks associated with formally seeking assistance. Furthermore, through applying Symbolic Interactionism, we see how participants have learned through social interaction and institutionalized expectations what “counseling” represents. Symbolically speaking, participants have learned through their interactions with others and institutions that “the counseling center” = “something on your transcript” = “unstable”. Through the Social Identity Theory we are able to demonstrate how participants compare their current level of distress to their expected self in the future as a nurse and elect to tolerate present day distress for protection of their future legitimacy.

***Subtheme 4.2: Informal Help as Safe Identity Space***

While some students sought help, these students’ help-seeking behaviors were almost always informal and dyadic and had parameters placed on their identities.

“I speak with my sister. Since she is not in nursing school, she is less likely to evaluate me differently than my classmates who will likely view me as different once they find out about my challenges. My sister listens. I would not feel comfortable telling my classmates about my anxieties and how I feel about being judged as anything less than ‘one of theirs’, so I can be ‘my sister’s sister’ instead.”

(Zahra, Level 8)

“I have a childhood friend who attended engineering school. She does not know anything about nursing; and that is exactly why I confide in her. She evaluates me according to no one else’s standard but her own. When speaking to her, I do not have to perform—I can just be.”

(Reem, Level 8)

Participants created a safe space separate from their nursing identity (Social Identity Theory), as they spoke with friends or family members who were not affiliated with nursing education. Participants chose to communicate their concerns with individuals outside of their nursing identity boundary in order to preserve the integrity of their nursing identity. Reem highlights the sense of escape from nursing specific evaluation criteria when speaking with a person not associated with nursing, illustrating those relationships outside of nursing provide refuge from professional identity surveillance. Using the Social Identity Theory, we are able to illustrate the interactional asymmetry of participants having a family member or friend listen to them without imposing interpretive frames based on nursing culture.

***Subtheme 4.3: Institutional Support as Final Option***

The vast majority of participants perceived formal institutional support as their very last option and sought formal help only when they felt their identity had been severely impacted and they were at risk of collapsing.

“… I failed clinically two times. I hadn’t slept in days. I experienced panic attacks before each clinical rotation. At some point I thought either I’d have to go ask for help, or pull out of school. Although I said to the university counselor that I was feeling stressed about finals, I think she knew what was going on here was a lot bigger than that.”

(Fatima, Level 4)

“I ended up seeing the counselor at the counseling center because my preceptor told me so. She said she feel that I am always exhausted so I go. The counsellor could tell right away something else was going on. I was terrified that this would be documented. But she assured me everything was kept private. It still took me going to the doctor because I had no other way out.”

(Maryam, Level 6)

When analyzing participant actions through the lens of the Identity Negotiation Theory, we see how participants weighed formal help-seeking behavior risks (risks of violating nursing identity norms) against non-help-seeking behavior risks (academic success risks) as well as how participants engaged in strategic partial disclosure when interacting formally with counselors (i.e., Fatima stating she was “stressed about finals,” Maryam saying she was “tired”) in order to protect their professional identity as much as possible. Furthermore, Maryam indicates how an acute crisis event (being told to go by preceptor) can overrule identity protective mechanisms but still demonstrates how participants will strategically minimize their reporting when necessary to maintain their professional status. Finally, the Identity Negotiation Theory allows us to demonstrate how participants re-write their accounts to portray identities consistent with societal expectations.

While the three lenses converged on students’ reluctance to access formal help, they differed regarding the reason. The Social Identity Theory explained that the students avoided formal help due to fear of breaching nursing identity norms. Symbolic Interactionism indicated that students came to understand institutional meanings through their experiences with others. The Identity Negotiation Theory suggested that students assessed potential harm to their future selves (identity projections) if they did or did not pursue formal help.

### 3.5. Theme 5: Reconciling Personal and Professional Identities

This overarching theme describes the developmental efforts of the study’s participants to create a coherent story about how they have connected their personal and professional experiences with their mental health; each individual’s success or failure differs.

***Subtheme 5.1: The “Future Nurse” Self***

Participants develop a projected future “self” and utilize their perceived identity of that future “self,” along with their present-day issues as motivation.

“Myself… I’m telling myself this is temporary. I’m not a mentally ill nurse… I’m a nursing student having a difficult time. In all reality, the true me (the future me) is the strong nurse. What I am going through right now is just… a short period of time. That I need to get through.”

(Maryam, Level 6)

“I carry around a picture of myself in my white coat on my phone. Every time I feel like I want to give up, I pull out my phone and look at it and tell myself that’s who I’m becoming. The anxiety is just a phase. The real me is that confident nurse in the photo.”

(Noura, Level 8)

To engage in future-self prototyping, students establish an idealized identity (“strong nurse”), and view current difficulties as deviations from this future self. Noura illustrates how tangible representations of a desired identity help provide a reminder of what she wants to become and serve as an anchor to maintain her while struggling through present adversity. Biographic Continuity Work is exemplified using Symbolic Interactionism. Students create a continuous narrative that unites their identities across time. Lastly, through the Identity Negotiation Theory, students are able to associate their current sacrifices with future rewards to enable them to persevere in the present.

***Subtheme 5.2: Integration* vs. *Separation***

There exists another aspect of the participants’ differences in terms of whether they strive to include their mental health experiences within their evolving nursing identity or if they attempt to totally segregate these parts of themselves.

“For such a long time, I thought I had to choose either I could be a nurse or I could be someone who suffers from anxiety… however, today I am trying to believe that I can be both. Being anxious doesn’t mean that I won’t be able to provide excellent patient care. Perhaps being anxious will even allow me to be more empathetic towards some of our patients.”

(Huda, Level 6)

“…in general, I try to keep them separated. Mental health is my own personal affairs. Nursing is my profession. These two areas do not cross paths. If they did, I would probably lose myself.”

(Bayan, Level 4)

The quotes listed above demonstrate an emerging distinction in identity negotiation techniques employed by the students. One student is attempting to combine her mental health experience with her nursing identity, and transform her mental health into a professional benefit (better ability to connect with patients). The other student is attempting to separate her mental health experience from her nursing identity by completely dividing the two domains. The Social Identity Theory explains the difference in terms of flexibility in prototypes; the first student has experienced or created a more inclusive prototype whereas the second continues to function under the old paradigm of separating the two identities. Symbolic Interactionism provides an explanation for the different processes used for constructing new stories for integration versus maintaining vigilant interactions when attempting to divide. The Identity Negotiation Theory explains the long-term costs associated with projecting one’s identity: integrating will likely produce increased cohesion but will necessitate continued risk taking; compartmentalization will immediately reduce stress but may lead to later negative consequences.

***Subtheme 5.3: Reframing Distress as Growth***

Some students report on how they tried to define their mental health distresses as an integral part of their development as professionals in nursing.

“When you look back now, I feel like this helped me to be a better nurse. I know what it is like to be completely overwhelmed. That’s why when a patient arrives who is anxious, I do not just learn about it… I see them because I’ve experienced those feelings too.”

(Huda, Level 6)

“I used to think that all patients complaining of anxiety were just weak. But before I had my own experience of depression, I understand that there is no doubt it is very real, very painful and takes a lot of courage to deal with it. I think this helps make me a more empathetic nurse than I might have been if I hadn’t had these experiences.”

(Fatima, Level 4)

These quotations exemplify the construction of a posttraumatic growth narrative (Symbolic Interactionism); the students are applying previous negative experience as positive assets for their current professional work. Huda’s description of how she transformed her mental health experience into an asset for understanding her patients’ pain demonstrates the transition from stigma to compassion, as direct experience dissolves previously held negative attitudes toward mentally ill patients. The Social Identity Theory explains how the student has reconceptualized her identity; she has shifted her mental health history from an out-group threat to an in-group resource. Identity Negotiation Theory accounts for the biographical continuity strategy applied by the student; she has incorporated past, present and future self-narratives together in order to provide coherence in developing her professional identity across different contexts.

The three lenses agree that the process of integrating mental health into the professional identity of nursing is a developmental issue; however, they differ regarding the pathway taken. The Social Identity Theory posits that changing group prototypes is the most important element in progressing toward a more inclusive professional identity; Symbolic Interactionism holds that reconstructing narratives is the primary process leading to professional advancement; and The Identity Negotiation Theory indicates that a deliberate change in the perceived costs and benefits along the educational trajectory represents the most important factor influencing the evolution toward a more coherent professional identity. The varying degrees of mental health integration among students (integrating vs. compartmentalizing) illustrate that both individual and situational factors affect whether individuals integrate their mental health experiences into their professional identity; and this is an aspect that can be explored more clearly using an integrated approach than either one lens alone.

***Converging Cases, Cross-Case Analysis, and Theoretical Contrasts***

There was a high level of cross-case agreement among the three broad assertions made in the analysis: (1) Mental health identity is formed through social and interactive methods. (2) Stigma significantly limits an expression of mental health identity and obtaining treatment for mental health problems. (3) The variation in contexts (i.e., the classroom and clinical environments) affected how students expressed their mental health identities. While the use of triangulation supported the study’s main assertion that mental health identity is socially and interactively constructed within nursing education, which would require a multidisciplinary approach to investigate, the cross-case analysis provided many theoretical contrasts to further develop the study’s integrative model.

***Agency* vs. *Structure in Creating Identities:***

Social Identity Theory and Symbolic Interactionism emphasize structural constraints to identification, from identification based upon group membership and inter/intra-group barriers to identification created by members’ actions and interactions. While the Identity Negotiation Theory emphasizes individual agency (strategic choices about identifying with specific groups/categories). The cases analyzed in this research illustrate that participants chose bounded agency; making strategic decisions under the influence of very strong group norms. Thus, the integrative model addresses the conflict between structure and agency by viewing agency as occurring within a socially created structural framework.

***Stability* vs. *Contingency of Group Prototypes:***

According to Social Identity Theory, there are relatively stable prototypes of groups that exist across most social situations. However, it is clear from the findings of this study that the prototypes used by the participants were highly malleable and variable when the participants acted in their classroom versus their clinical settings. Therefore, the Identity Negotiation Theory provides a resolution to this conflict by explicitly including variability across social situations as a critical aspect of its prototype concept. This allows for the possibility that prototypes may be contingent upon the situation.

***Individual* vs. *Relational Levels of Analysis:***

Symbolic Interactionism is primarily concerned with dyadic processes of meaning creation. In contrast, the Social Identity Theory is fundamentally concerned with processes at the group level. While the data collected in this study indicated that both dyadic processes of creating meaning and processes at the group level were crucial elements in how participants constructed their identities, they occurred concurrently. More specifically, while the dyadic processes were reinforcing the norms at the group level, the group-level norms were constraining what could occur at the dyadic level. Therefore, the integrative model contains these two levels of analysis in a dialectical relationship rather than prioritizing one over the other.

## 4. Discussion

This study employed Interpretative Phenomenological Analysis to explore how nursing students at King Khalid University developed, negotiated, and enacted their mental health identities within their profession through education. Additionally, this study provides connections to previous research, offers new theoretical frameworks and identifies recommendations for nursing education policy and practice.

### 4.1. Navigating the Stigma Boundary

***The “Strong Nurse” Imperative***

Findings from the current study align with previous studies on stigma in health professions education. Previous research has shown that many student nurses hold negative views regarding stress and help-seeking. Fear of disclosure and fear of being perceived as unable to perform their role were the main reasons for not seeking help. Most participants in the current study revealed their mental health issues to family members or friends outside of nursing. Some selectively revealed their issues to trusted nursing peers (i.e., roommates or peers at the same level). This established micro-safe spaces throughout the cohort. Broader disclosure remained out of bounds.

Collectively, these findings suggest that the “strong nurse” prototype functions as a strong identity boundary. This prototype is the ability to be emotionally resilient without demonstrating distress. It defines who belongs to the student nurse group (Tajfel & Turner, 1979; Haslam et al., 2009).

This study extends prior research by identifying the interactional processes involved in enforcing this prototype. Participants noticed changes in how peers looked at them after revealing their mental health issues. This exemplifies the looking-glass self-dynamic. It is fundamental to symbolic interactionism (Mead, 1934). Similar findings were noted by Sukhera et al. (2022). They indicated that an emancipatory help-seeking discourse in medical education conflicts with a performance discourse. This performance discourse promotes perfectionism. Both discourses functioned as surveillance for participants. They forced participants to internalize expectations of judgment as self-surveillance. Global, moderate to high levels of mental health stigma exist among nursing students. Recent integrative reviews confirm this. Such stigma manifests as negative attitudes towards patients. It also appears as internalized shame directed toward students themselves (Wang et al., 2026).

**Strategic silence as a means of maintaining identity**

Participants’ intentional nondisclosure represents a valid means of maintaining their identity (Ting-Toomey, 2005). Student nurses utilized strategic silence to manage their identity even in supportive environments. This created a continued sense of identity. The cost of disclosure exceeded the value gained. This study challenges the simplistic conceptualization of the help-seeking/help-avoidance dichotomy in nursing education (Galbraith et al., 2014; Mitchell, 2018). Participants’ avoidance of help did not necessarily reflect an unwillingness to seek assistance. Instead, it demonstrated a need to protect their competent student nurse identity. This protection came at great psychological cost. Recent systematic reviews and meta-analyses of educational interventions indicate that “confidentiality concerns and perfectionism have been identified as obstacles…” with a barriers-focused source (Alsalman et al., 2024). These prevent healthcare students from obtaining professional mental healthcare.

The implications for designing intervention programs are substantial. Silence can be strategic rather than uninformed. Awareness campaign efforts will likely fail. Students already know what resources are available. They require a safe pathway to access those resources. This pathway must not compromise their image as professionals.

Scholars like Moxham et al. (2016) demonstrated that immersion-based clinical placements (i.e., Recovery Camp) reduced stigmatizing attitudes toward individuals with mental illness. However, the present study indicates that such placements do not negate identity-based barriers to disclosing personal struggles.

### 4.2. Theme 2: Developing the “Mental Health” Label

***A Medicalization* vs. *A Normalization Perspective***

Student respondents identified their distress in various manners. Some utilized a medically based label (“anxiety”). Others provided a normalization-based response (“just stressed”). These responses provide evidence of the symbolic-political processes associated with the formation of student mental health identities. Sukhera et al. (2022) demonstrated that competing discourses regarding mental illness exist. However, they did not illustrate how students utilize those discourses to construct their own mental health identity. The current research illustrated how self-labeling can be used strategically. It can also protect one’s identity.

Student respondents identifying themselves as “just stressed” protect their agency. This label does not align with a medically based definition of anxiety. It defines their distress as temporary and context-specific. Students who identify themselves using a medically based label (“I have anxiety”) create a biologically based explanation for their distress. This affords them compassion. However, they also accept the risks of stigma associated with such a label. This illustrates the duality of diagnosis in the sociological literature. It is liberatory, yet restrictive (Haslam et al., 2009).

This variability has been explained within a Social Identity Theory framework. Specifically, participants are making prototype comparisons. They assess whether a self-label will fit into acceptable in-group categories. The categorization of “stressed” is identity-safe. It is viewed as a normalized aspect of nursing culture. The categorizations of “depression” and “anxiety” would deviate from the category of “the strong nurse.” This requires caution when incorporating them into the participant’s identity.

From a Symbolic Interactionism perspective, labels are not permanent constructs. They are developed through peer interaction, faculty modeling, and institutional messaging. They are subsequently used strategically in self-presentation.

**The Intensely Self-Monitoring Process of Nursing Students**

Students described their intensely self-monitoring process (“I have two versions of myself”) as representing the panopticon model of self-regulation (Foucault, 1975). The internally performed institutional gaze becomes an internalized self-gaze. This provides protection for the performing self. Mitchell (2018) found that nursing students experienced significant obstacles related to seeking help for their mental health issues. The current research demonstrates that those same obstacles occur at the conscious level. They operate through micro-processes. They also operate at the macro-institutional level.

As defined by the Identity Negotiation Theory, the cost of this “two-selves” approach is substantial. Maintaining the performance of roles across different contexts results in exhaustion (Ting-Toomey, 2005). It also produces feelings of alienation and a lack of authenticity. Long-term utilization could negatively affect students’ mental health. It could also affect their ability to grow professionally.

For example, Reem (Level 8) describes engaging in a pre-clinical ritual. She practices her facial expressions in front of the mirror. She checks for hand tremors. This represents an extreme version of the self-surveillance model. In cultures where public displays of emotion by females are strictly controlled, nursing students enter professional identity development with existing skills to emotionally hide themselves. The “mask” becomes an additional layer of culturally learned gendered self-presentation.

As a result, labeling occurs on three levels. These are individual (cognitive), interpersonal (interactional), and environmental (structural). This supports interventions that address identity-related barriers to accessing help. Such barriers go beyond information and attitude obstacles.

### 4.3. Theme 3: The Clinical–Classroom Divide

**Contextual Identity Salience and the Bedside as a High-Stakes Identity Arena**

Patterson et al. (2017) reported that mental health placements increased students’ confidence. The current study demonstrates that all types of clinical experiences increase pressure on students to perform their identities. This is especially true when students have their own mental health issues. In particular, the bedside represents a high-stakes identity arena. It is here that the ‘Competent Nurse’ prototype is most salient and heavily scrutinized. The stakes are higher. More people are watching. The consequences of being unable to meet expectations are greater than anywhere else. Students quickly move between their ‘Student’ identity and their ‘Clinical Nurse’ identity.

**The ‘Student’ identity allows vulnerability. The ‘Clinical Nurse’ identity requires competency**. This movement creates a high level of identity threat (Ting-Toomey, 2005). Nursing programs prepare students with skills prior to clinical placement. However, they do not prepare students for the identity work required during transitions between academic and clinical settings. Labrague (2024) conducted a systematic review on the emotional challenges encountered by student nurses during clinical placement. Many students encounter traumatic events in clinical placement. These include witnessing patient death, caring for critically ill patients, and exposure to hostile behaviors. Labrague (2024) reports that 60–70% of students encounter some form of distressing event during training. Moreover, Labrague (2024) reports that lack of support from clinical facilitators and experienced nurses forces students to face emotional challenges independently. This results in decreased personal and professional development. A PTSD screen administered to student nurses yielded positive results (range of 15% to 53.3%). This demonstrates that students in clinical education are likely exposed to significant trauma and stress (Kratovic et al., 2021). Together, these studies provide evidence that the clinical educational environment is not simply a place for developing technical skills. It is a place for testing identity. It is also a place for regulating emotions in an environment that continually monitors and polices emotional expression.

#### The Transition as an Identity Threat

Theoretically, the major outcome of this study was that the type of environment did not cause identity confusion. Instead, the rapidity of transition caused it. Students were confused regarding their true identity after switching rapidly between two distinct environments. This indicates identity fragmentation. According to the Identity Negotiation Theory (Ting-Toomey, 2005), fragmentation is created by the number of times an individual switches between environments. It is also created by how fast they switch. What happens within those environments matters less. Therefore, the alternating classroom–clinical format of nursing education may maintain identity ambiguity for students with mental health concerns. This study expands upon previous research on placement outcomes (Moxham et al., 2016; Patterson et al., 2017). It illustrates that the same person develops and enacts vastly different identities within two structurally similar environments.

### 4.4. Theme 4: Help-Seeking as Identity Betrayal

This study identifies institutional barriers contributing to students’ perceptions of formal help-seeking. Students view it as a potential threat to their professional identity. Galbraith et al. (2014) and Mitchell (2018) previously identified students’ fears regarding the professional implications of disclosing emotional problems. However, previous research has not examined how students develop such perceptions. Participants described three mechanisms they believed could operate: the existence of a counseling record, possible employer access to records, and the irreversibility of disclosure. These accounts demonstrate perceived institutional risk. Because the study did not audit university counseling-record practices or employer screening rules, we cannot verify whether such access occurs; the finding is that students believed disclosure could be consequential and managed their help-seeking accordingly.

Recent studies on mental health in higher education confirm that many healthcare students are concerned about seeking counseling services. They fear being punished or disciplined by their institution. They also fear that counseling records will appear in their academic records (Alsalman et al., 2024).

Social Identity Theory provides a framework for understanding why students believe that formal help-seeking creates a permanent record. This record signals deviation from the “strong nurse” prototype. Specifically, students believe that formal help-seeking signals non-conformity to the “strong nurse” prototype. This results in legitimacy loss. Symbolic Interactionism provides a lens for understanding how students construct meaning related to help-seeking. This occurs through interactions with faculty, peers, and other institutional agents. The Identity Negotiation Theory explains how students weigh short-term benefits of obtaining help against long-term identity damage (Ting-Toomey, 2005).

The creation of informal dyadic help was an adaptive strategy. Participants created safe zones outside of nursing. They disclosed personal issues without risking their professional reputation. This supports prior research demonstrating that support exists beyond formal sources (e.g., support groups). However, the current research extends prior work. It identifies both the importance of availability and the source of support. Students identify that outside support reduces identity risks. This support comes from family members or friends outside of nursing. However, there are significant drawbacks to informal support. Informal supporters often lack clinical knowledge to address serious mental health crises. Secrecy associated with informal support can strain relationships. Furthermore, limiting disclosure within professional settings limits opportunities to create an integrated professional identity. This identity should include self-care behaviors modeled during times of crisis.

Participants reported preferring support from family members outside of nursing. This preference reflects cultural norms for confiding in family rather than formal authorities. Family centeredness can serve as a barrier to help-seeking. This occurs when family members subscribe to negative stereotypes regarding mental illness. The data indicate that participants selected family members outside of nursing as their primary sources of support. Family support appears less risky than institutional support. However, the most important factor is selecting sources who do not impose nursing-specific evaluative standards.

### 4.5. Theme 5: Reconciling Personal and Professional Identities

***The Dilemma of Integration* vs. *Separation***

Students’ choices between integration (“I can be both”) and compartmentalization (“I keep them separate”) illustrate the differing levels of development in how they negotiate their identities. Integration facilitates true professional empathy, yet it necessitates constant risk-taking of their identity. Compartmentalization gives students instant peace-of-mind; however, it creates an environment of potential psychological pressure, and limits a nurse’s ability to truly practice professionally. Based on social identity theory, integrators develop a new prototype that incorporates the idea of being vulnerable while compartmentalizers continue to hold onto the original “the strong nurse” model. Integration requires that students construct a new narrative regarding themselves; compartmentalization requires that students remain vigilant about the interactions between these two aspects of their lives. According to identity negotiation theory, there is a trade off when using either method—integrators achieve cohesion by constantly negotiating their identity; compartmentalizers immediately feel comfortable, yet, they delay the cost of ultimately creating a potentially problematic relationship between their student self and professional self (Ting-Toomey, 2005).

Huda (Level 6) and Bayan (Level 4), representing different levels of experience, illustrate the extremes of responding to collective experience. Huda’s developing integration (“today I am working on believing I can be both”), shows that over time nursing education could allow some students to expand their sense of identity. Bayan’s persistent compartmentalization (“these two areas do not cross paths”) may serve as a defensive mechanism because nursing continues to be limited as a choice of profession and the threat of losing professional standing has severe implications. Programs should not require students to take either path but rather provide opportunities for integration, such as through guided peer support, narrative reflection and mentors disclosing their own vulnerabilities to reduce the identity risks of being authentic.

#### Turning Liabilities into Assets

Perhaps the most hopeful finding was that many participants were able to transform previous liabilities into assets by describing their difficulties as positive learning experiences (“This made me a better nurse”). In terms of social identity theory, this represents identity reconstruction: students’ previous histories of mental illness transition from an out-group threat to an in-group asset, thus expanding the prototype of competent nurse to include empathy derived from lived-experiences. Using Mead’s (1934) “I” and “me”, participants are able to reconstruct their past self and establish a cohesive narrative that produces intercontextual coherence throughout their educational experiences in classrooms, clinical settings and personal lives (Ting-Toomey, 2005).

When Fatima (Level 4) said she believes her experience allows her to see the individuality in each patient experiencing anxiety and Huda (Level 6) shifted her perception from viewing individuals with anxiety as weak to viewing them as having courage demonstrates what Tedeschi and Calhoun (2004) refer to as post-traumatic growth. Research on post-traumatic growth among nursing students found that resilience correlated positively with post-traumatic growth (r = 0.54, *p* < 0.01), whereas burnout correlated negatively with post-traumatic growth (r = −0.35, *p* < 0.01); resilience, secondary traumatic stress, and burnout were all independently associated with post-traumatic growth in multiple regression analysis (Alkrenawi et al., 2025). While stigma creates large obstacles for students integrating their identities, those students who successfully navigate these obstacles may emerge with enhanced professional skills.

In addition to orienting current struggles toward a future “nurse” self (“I am a student facing hard times right now who will become a confident nurse”), participants showed evidence of a temporal component to identity formation. Aspirational identification allows students to endure through the present challenges with the belief that the costs will translate into benefits in the future (Haslam et al., 2009; Ting-Toomey, 2005). Noura’s (Level 8) reference to a photo of herself in her white coat as an aspirational anchor illustrates how tangible representations of professional identity can help provide students with a source of inspiration during difficult periods in school. This is consistent with Markus and Nurius’s (1986) conceptualization of possible selves—future-oriented self-representations influence present behaviors and emotions.

Career counselors and mentors can assist students in projecting their future-selves though, if the prototypes fail (i.e., academically failing or withdrawing), students may lose significant amounts of psychological capital.

**Theory Development**

Mental health identity development in nursing education is a continuously ongoing process of negotiating with one’s environment. Using the Social Identity Theory (prototype development), Symbolic Interactionism (narrative reconstruction), and Identity Negotiation Theory (strategic path selection) to develop an integrated model that explains how students can use multiple paths to achieve their mental health reconciliation through time. This provides a new perspective on student mental health by transforming it from a deficit-based model into an integrated model for developing student identities. As all three theories have identified limitations when used alone—the Social Identity Theory does not explain individual variation; Symbolic Interactionism leaves out structural factors; and the Identity Negotiation Theory may over-emphasize individual choice (Ting-Toomey, 2005); using only one lens would likely hide the contradictions found in the study results (agency—structure, contingency stability, relational individual).

This study has provided contextual evidence that demonstrates cultural and institutional forces shape specific forms that tension occurs within identities. The “bounded agency” documented in this study operates within fields that are both normatively professional and also religious, gendered, and familial. Therefore, the integrated model developed here is applicable beyond the immediate setting to any other setting where professional identity construction intersects with cultural norms that stigmatize psychological vulnerability.

### 4.6. Practical Applications

***Development of curriculum content:***

- Reduce the stigma at the level of identity by providing interventions against the prototype of “strong nurse”. That is, faculty modeling vulnerable behavior as competent professional behavior (Sukhera et al., 2022). In order to do so, it is necessary to consider religion and culture: framing vulnerability within traditions of compassion and human fallibility may be more culturally relevant than secular psychology frameworks.
- Provide supportive systems during classroom-to-clinical transitions that allow students to engage in identity work via peer-support groups and reflective practice. Considering the segregated-by-gender nature of many nursing education programs, same-sex peer support groups can leverage existing cultural norms of female solidarity.
- Establish opportunities for students to develop narrative-based, integrated professional identities that frame mental health experience as positive contributions. Examples may include inviting nurses who have successfully managed mental health challenges to share their experiences, providing culturally and religiously grounded role models.
- Include emotional regulation training within nursing curriculums. Recent experimental research demonstrated that an eight-session program based upon the Gross model significantly improved emotional adjustment and reduced psychological distress among nursing students (*p* < 0.001), with effect sizes indicating substantial clinical relevance (Balideh et al., 2025).

**Policy creation/implementation at institution level:**

- Protect counseling records from academic and employment files, and clearly communicate to students that counseling sessions are protected from disclosure; physician-specific reforms have been proposed (Saddawi-Konefka et al., 2025); similarly needed reform exists in nursing. This requires explicit policy statement at ministry level to ensure counseling confidentiality is maintained across institutional boundaries.
- Build peer-support systems providing safe spaces for disclosure in group settings. These should be designed to complement rather than replace family support systems recognizing the cultural primacy of family in many societies.
- Educate clinical instructors and preceptors to respond to student distress without inducing identity threat. Due to the high proportion of expatriate nursing faculty in some regions, training should address cross-cultural sensitivity in responding to students’ concerns regarding identity.

## 5. Future Research Needs

- Longitudinal tracking of identity negotiations over time, particularly through transition periods from student to registered nurse in the employment system.
- Comparative analysis of identity constructions of mental health across cultures, comparing findings of this study to those conducted with nursing students in other Gulf Cooperation Council countries and in Western contexts.
- Design and evaluation of interventions targeting identity-level barriers to help-seeking. Particular attention should be paid to approaches that are culturally adaptable.

### Strengths and Limitations

This study presents several strengths. First and foremost, this study is the first to apply an Integrated Model of Qualitative Analysis using three theoretical models. Therefore, the inclusion of multiple theoretical paradigms into a single IPA model represents an advancement in qualitative analysis methodology in healthcare education. Additionally, the incorporation of both idiographic (context-specific) and nomothetic (patterns across cases) elements along with researcher reflexivity and trustworthiness criteria contribute to the credibility and dependability of the findings produced by this study.

Researchers also recognized several potential weaknesses associated with their qualitative investigation. Because all data collection occurred at a single location (King Khalid University), the transferability of the results to other cultures or educational settings may be restricted. Specifically, the institutional culture which includes aspects such as faculty demographics, clinical placements, and policies surrounding the university counseling center may vary greatly from those found at institutions located in other locations or countries. Further, as the primary means for verifying student perceptions of their mental health was through their own accounts, labels of mental illness experiences assigned by participants were not validated through interaction with clinical staff. Although the number of participants included in this study supported an IPA, the idiographic focus of IPA precludes generalization, though transferability is enhanced through thick description.

The gender homogeneity among participants (i.e., all participants were females) may result in less understanding of male nursing students’ experiences. However, the gender homogeneity is reflective of the realities experienced by nursing education in Saudi Arabia, where virtually all nursing education programs continue to be primarily composed of females. Therefore, although the lack of male voices is a limitation of this study, it is also reflective of the current demographic makeup of practicing nurses in many areas who are predominately female. Thus, future investigations should address whether male nursing students who likely experience a different type of stigma for entering a traditionally feminized profession experience similar or differing identity negotiation processes.

Furthermore, researchers attempted to use reflexive bracketing to reduce educator bias during participants’ responses about potentially sensitive topics; however, it cannot be ruled out that being educators may have impacted participants’ responses about sensitive topics.

Student accounts of counseling-record and employer-access risks were perceptions reported in interviews. We did not audit counseling-record policies or employer recruitment procedures, so the findings should not be read as confirmation that such access occurs.

Lastly, because participants self-selected into a study about mental health experiences, students who were most distressed or least comfortable discussing mental health may be underrepresented; the sample may therefore reflect students with greater readiness to disclose.

## 6. Conclusions

Developing a mental health identity in nursing students is a dynamic, socially constructed process shaped by multiple contexts. This study synthesizes three theories—Social Identity Theory, Symbolic Interactionism, and Identity Negotiation Theory—to describe how nursing students construct meanings around mental health experiences, explain how students negotiate stigma across social transitions, develop coping strategies, and integrate a professional self-identity that encompasses both competent nurse and individual experiencing psychological distress.

Contextual influences may operate distinctively in this setting, where professional nursing standards intersect with Islamic values, gender-segregated education, family-centered social structures, and government-controlled employment systems. The ‘strong nurse’ prototype may acquire religious significance—as one participant put it, distress could mean that ‘you fail a spiritual test’ (Maryam, Level 6)—strategic silence functioned as a means of identity self-protection, the contrast between clinical and classroom settings shaped where distress could be expressed, and perceived, though unverified, employment-related risks discouraged access to institutional support. Because these cultural-level interpretations were not directly tested and rest on a small, single-site, all-female sample, they should be read as tentative and participant-anchored rather than as confirmed institutional facts.

These findings challenge deficit-based views of mental health in nursing students. Individual-focused interventions alone are insufficient; structural approaches that reduce disclosure costs, broaden competency definitions to include vulnerability, and scaffold integrated professional identity development offer viable alternatives. Such interventions must be culturally grounded, working with existing religio-cultural and familial values while addressing gendered disclosure constraints, to foster authentic professional identities. Such theory advances understanding of mental health identity complexity among nursing students and informs educational environments that support genuine professional growth and psychological wellness.

## Figures and Tables

**Table 1:** Anonymized demographics of the participants. *N* = 9.

Pseudonym	BSN Level/Year	Clinical Experience Category/Rotation
Aisha	Level 4	6
Bayan	Level 4	6
Fatima	Level 4	8
Huda	Level 6	8
Maryam	Level 6	8
Noura	Level 8	18
Reem	Level 8	18
Zahra	Level 8	18
Maha	Level 8	18

**Table 2:** Combined display of superordinate themes and their corresponding theoretical maps. Theme and Sub-themes.

	Social Identity Theory (SIT) Constructs	Symbolic Interactionism Constructs	Identity Negotiation Lens Constructs	Cross-Convergence
1. Navigating the Stigma Boundary	In-group prototype Out-group threat	Looking-glass self (Mead) Meaning-making through peer observation and unstated judgments Interpretive repertoires of heroism	Identity coherence across contexts Legitimacy strategies Role expectation management (student, future nurse, peer)	All three lenses confirm stigma as the central force shaping mental health identity construction. Tension exists regarding locus of agency (structural vs. individual).
1.1 The “Strong Nurse” Imperative				
1.2 Silence as Self-Protection				
1.3 Selective Disclosure				
2. Developing the “Mental Health” Label	Self-categorization processes Group prototype comparison	Symbolic meaning-making Narrative identity construction Generalized other internalisation Panopticon self-regulation	Cross-contextual label consistency/inconsistency Split-self process “Experiencing self” vs. “performing self”	SIT and Symbolic Interactionism converge on social construction of mental health categories. INT introduces tension by revealing context-dependent label variation.
2.1 Medical vs. Moral Framing				
2.2 Self-Surveillance and Monitoring				
3. The Contextual Clinical–Classroom Divide	Contextual identity salience Normative group boundaries In-group prototype prominence by setting	Situational self-presentation (Goffman, 1959) Frontstage/backstage behavior Biographic disruption Existential confusion	Role transition management Identity continuity/discontinuity Cross-contextual legitimacy requirements Rapid identity switching	INT provides primary explanatory power for contextual variability.
3.1 Performing Competence at the Bedside				
3.2 Vulnerability in the Classroom				
3.3 Identity Fragmentation During Transition				
4. Help-Seeking as Identity Betrayal	In-group betrayal anxiety Normative conformity pressure Intergenerational stigma transmission	Institutional meaning-making through social interaction Learned symbolism of “counseling” Peer narrative influence	Disclosure cost–benefit analysis Long-term identity projection Legitimacy maintenance Strategic partial disclosure Crisis-threshold decision-making	All three lenses converge on formal help-avoidance.
4.1 Perceived Threats to Future Professional Suitability				
4.2 Informal Help as Safe Identity Space				
4.3 Institutional Support as Final Option				
5. Reconciling Personal and Professional Identities	Identity integration vs. compartmentalization Future self-prototyping Evolving group prototypes	Biographical integration Narrative reconstruction of identity Posttraumatic growth narrative “I” and “Me” reconstruction	Long-term identity projection Cross-temporal self-consistency Strategic shift in perceived costs/benefits Aspirational identification	Convergence on integration as developmental challenge.
5.1 The “Future Nurse” Evolving Self				
5.2 Integration vs. Separation				
5.3 Reframing Distress as Growth				

## Data Availability

The data presented in this study are available on request from the corresponding author.
